# Assessment of Human Health Risks Posed by Nano-and Microplastics Is Currently Not Feasible

**DOI:** 10.3390/ijerph17238832

**Published:** 2020-11-27

**Authors:** Andreas Brachner, Despina Fragouli, Iola F. Duarte, Patricia M. A. Farias, Sofia Dembski, Manosij Ghosh, Ivan Barisic, Daniela Zdzieblo, Jeroen Vanoirbeek, Philipp Schwabl, Winfried Neuhaus

**Affiliations:** 1 Competence Unit Molecular Diagnostics, Austrian Institute of Technology GmbH, 1210 Vienna, Austria; ivan.barisic@ait.ac.at; 2 Smart Materials, Istituto Italiano di Tecnologia, via Morego 30, 16163 Genova, Italy; Despina.Fragouli@iit.it; 3 CICECO-Aveiro Institute of Materials, Department of Chemistry, University of Aveiro, 3810-193 Aveiro, Portugal; ioladuarte@ua.pt; 4 PHORNANO Holding GmbH, 2100 Korneuburg, Austria; patricia.farias@phornano.com; 5 Programa de Pos-graduacao em Ciencia de Materiais, Departamento de Biofisica e Radiobiologia, Universidade Federal de Pernambuco-UFPE, Recife 50670-901, Brazil; 6 Fraunhofer Translational Center Regenerative Therapies TLC-RT, 97070 Würzburg, Germany; sofia.dembski@isc.fraunhofer.de (S.D.); daniela.zdzieblo@isc.fraunhofer.de (D.Z.); 7 Chair of Tissue Engineering and Regenerative Medicine, University Hospital, 97070 Würzburg, Germany; 8 Department of Public Health and Primary Care Centre for Environment and Health Herestraat 49 (O&N 706), KU Leuven, B-3000 Leuven, Belgium; manosij.ghosh@kuleuven.be (M.G.); jeroen.vanoirbeek@kuleuven.be (J.V.); 9 Division of Gastroenterology and Hepatology, Department of Medicine III, Medical University of Vienna, 1090 Vienna, Austria; philipp.schwabl@meduniwien.ac.at

**Keywords:** nanoplastics, nanoparticles, microplastics, microparticles, human exposure, biological barriers, biofilm, microbe carrier, toxicity, neurotoxicity

## Abstract

The exposure of humans to nano-and microplastic particles (NMPs) is an issue recognized as a potential health hazard by scientists, authorities, politics, non-governmental organizations and the general public. The concentration of NMPs in the environment is increasing concomitantly with global plastic production and the usage of plastic materials. NMPs are detectable in numerous aquatic organisms and also in human samples, therefore necessitating a risk assessment of NMPs for human health. So far, a comprehensive risk assessment of NMPs is hampered by limited availability of appropriate reference materials, analytical obstacles and a lack of definitions and standardized study designs. Most studies conducted so far used polystyrene (PS) spheres as a matter of availability, although this polymer type accounts for only about 7% of total plastic production. Differently sized particles, different concentration and incubation times, and various biological models have been used, yielding hardly comparable data sets. Crucial physico-chemical properties of NMPs such as surface (charge, polarity, chemical reactivity), supplemented additives and adsorbed chemicals have been widely excluded from studies, although in particular the surface of NMPs determines the interaction with cellular membranes. In this manuscript we give an overview about the critical parameters which should be considered when performing risk assessments of NMPs, including novel reference materials, taking into account surface modifications (e.g., reflecting weathering processes), and the possible role of NMPs as a substrate and/or carrier for (pathogenic) microbes. Moreover, we make suggestions for biological model systems to evaluate immediate toxicity, long-term effects and the potential of NMPs to cross biological barriers. We are convinced that standardized reference materials and experimental parameters along with technical innovations in (nano)-particle sampling and analytics are a prerequisite for the successful realization of conclusive human health risk assessments of NMPs.

## 1. Introduction

Nano-and microplastic particles (NMPs), nowadays omnipresent in the environment, originate from degradation processes of macroplastic waste in nature, textile- and tyre wear, products containing intentionally added NMPs (e.g., personal care products, household cleaning agents), and are released by plastic packaging material (e.g., bottled water, food wrapped in foils). Microplastic particles are well documented for entering the food chain via marine organisms, especially shellfish [1], but are also present in drinking water [2], table salt [3], fruit and vegetables [4], and other human aliments [5], all over the world. Li et al. calculated in a recent study that the exposure of infants fed with milk prepared in usual polypropylene baby bottles can reach up to 3 million microparticles per day [6].

Air pollution is a severe and well-known human health threat, especially in densely populated areas. NMPs contribute to airborne particulate matter PM10, which are directly inhaled by exposed persons or accumulate on surfaces or in the soil due to atmospheric fall out, entering surface and ground water reservoirs [7,8].

Overall, this brief glimpse on the various sources and appearances of NMPs in our environment illustrates the potential exposure of humans to NMPs in daily life.

Over the past years this perpetual and annually increasing (as plastic production and usage increases) human exposure to NMPs gained scientific and public attention, being recognized by political authorities such as the European Commission, European Chemicals Agency (ECHA) and the World Health Organization (WHO) as potential health hazard for man and wildlife [5,9,10,11,12,13,14,15,16,17].

Several studies have been conducted to assess the impact of (mainly) microplastic particles on different levels, including physico-chemical analyses describing parameters such as stability under various environmental conditions [18], chemical properties such as surface charge, and adsorption or leaching of chemicals [19,20,21], and biological effects on cellular, organismal and ecosystems level [22,23,24].

The research field on the biological effects of micro- and, in particular, nanoplastic particles is in its infancy, hence methodologies and procedures have not consolidated yet. In particular, the currently available analytical techniques are still lagging behind the urgent need to characterize particles <100 nm, although there have been some recent advancements in the fields of correlative microscopy and Raman imaging [25,26,27]. In sum, we have identified in a PubMed survey (search date 9 November 2020; search terms nanoplastic and human) only 11 articles dealing with the biological effects of nanoplastics [28,29,30,31,32,33,34,35,36,37,38], underlining the current lack of knowledge in this field. Together with the limitations listed in the following paragraphs, we come to the conclusion that an overall estimation of the health impact of NMP exposure remains practically impossible at the moment.

## 2. Limitations in Reference Materials

(a) *Reference materials reflecting the diversity of industrially produced polymers and mimicking natural degradation processes*. The vast diversity of NMPs present in the environment, regarding physical and chemical properties, is contrasted by the majority of studies using microparticles (in fact, spheres) made of a single plastic type, polystyrene (PS), which is not even among the top five globally produced polymers. Polyethylene (PE), polyethylene terephthalate (PET), polypropylene (PP) and polyurethane (PU) were used only in single studies so far [32,38,39,40], whereas none (using a mammalian model system) has been conducted using polyvinylchloride (PVC) NMPs. It remains highly questionable whether effects observed with PS microparticles can be extrapolated to NMPs made of other polymers. In addition, most of the studies so far focused on plastic particles with a size >1 µm, but nanoplastic (<100 nm) showed very different (physical and biological) effects, mainly due to their different surface to volume ratio. This is probably due to the limited availability of reference nanoplastic particles and more intricate analytical methods for their detection and characterization.

Environmental weathering and degradation processes lead to secondary NMPs with fundamentally different physico-chemical particle properties (such as surface charge, wettability, sorption/desorption capacity), which are neglected when pristine, primary micro- or nanoplastic particles are used for studies [41]. Industrially synthesized particles are usually non-polar, with a hydrophobic surface. Weathering and aging of plastics introduce oxygen-containing functional groups, which increase the polarity of NMPs, and thereby alter their interaction with other chemicals, organic matter and living cells [42]. A promising new approach to generate NMPs, which is based on laser ablation of plastic foils in aqueous solution, yields particles with surface modifications similar to “nature-like” weathering [32]. A further benefit of this method is that it could be extended to plastic types other than PS and PET, covering all abundantly present polymer types. Colonization of microparticles and biofilm formation by bacteria impact on the degradation process of plastics [43,44]. The fact that NMPs can enter the human body via two main routes, inhalation or ingestion, adds another level of complexity, as NMPs come into contact with mucus (airway) or saliva, low pH gastric fluid and digestive fluids in the intestines, which may further act on the particle’s surface [45].

(b) *Reference materials to tackle the role of NMPs as carriers of chemicals (additives, adsorbed pollutants) and microbes (biofilm)*. With regard to the analysis of the biological effects of NMP exposure, it has to be considered that plastic material contains chemicals such as plasticizers, pigments, flame retardants, fillers or stabilizers [46]. Nowadays, thousands of different chemicals are currently used for these purposes, and it is known that some of these chemicals can leach out during the product life cycle into the environment, leading to endocrine disruption or acute toxicity in exposed organisms [47]. Another hardly investigated aspect concerns the sorption of (in) organic pollutants and heavy metals when NMPs circulate in the environment and the potential effects upon uptake in organisms [42,48,49]. Ultraviolet (UV) weathering of plastics enhances the release of additives which exert biological effects in a mammalian cell-based assay, although when applied at concentrations >1000× higher than measured in environmental samples [19]. Some studies mimic natural leaching e.g., by soaking microplastics in saline solution [50], while other studies used organic solvents such as hexane or dichloromethane to extract hydrophobic additives (e.g., bisphenol A (BPA)) from plastics and solubilize adsorbed chemicals (e.g., PCB) to determine the leaching of chemicals from NMPs [51,52]. Extractions using organic solvents yield data hard to compare to nature-like conditions. Overall, the contribution of NMPs as carriers of harmful chemicals and pollutants might be negligible. In a worst-case scenario the European Food Safety Authority (EFSA) estimates that the increase of the total human exposure towards harmful pollutants caused by microplastics in food is minimal: polychlorinated bisphenyls (PCBs) contribute to the overall exposure <0.006%, polycyclic aromatic hydrocarbons (PAHs) <0.004% and BPA <2% [53]. Finally, there is hardly any standardized NMP material available that contains precisely defined amounts of chemical additives to be tested (e.g., only two CRM products for phthalates) in order to be able to investigate the leaching out of these chemicals [54]. The situation is similar for organic pollutants and adsorbed heavy metals. Nonetheless, NMPs may exert harmful effects by scavenging metal ions in aquatic ecosystems as well as in soil, thereby reducing their bioavailability for microbes and plants [49,55]. Regardless of this, the toxicity of chemicals is already determined separately, e.g., by the European Registration, Evaluation, Authorisation and Restriction of Chemicals (REACH) regulation.

## 3. Limitations in Analytical Methodology

Currently, the analysis of NMPs in environmental or biological samples is laborious, requiring great instrumental efforts and is not suitable to collect high-throughput, quantitative data. A specific challenge for any kind of NMP analysis is the collection and processing of samples, given the fact that NMPs are omnipresent in the air, and most lab equipment is made of or contains plastic material, which leads to sample contamination.

An ideal method for NMP detection and characterization should yield quantitative data on particle concentration and qualitative data on particle size, structure and chemical composition [56].

Methods such as dynamic light scattering (DLS), nano-particle tracking analysis (NTA) or high-resolution microscopy are well established for determining particle size and structure, but it remains very challenging/laborious to perform quantitative measurements of particle concentration in biological samples and to determine the chemical identity of particles. Micro Fourier-transform infrared spectroscopy (µFITR) and micro Raman spectroscopy are the most frequently used techniques for particle identification, but both methods are technically limited regarding size (µFITR can be used for particles >20 µm only, µRaman works down to sizes of approximately 1 µm) and are not suitable for the analysis of large particle numbers [56,57]. Hence, at the current technological level, identification of nanoparticles (≤100 nm) in environmental or biological samples is not possible.

As mentioned above, a critical point concerning every NMP study is the potential contamination of samples with NMPs present in laboratory air or released by the (plastic) equipment used during collection and processing [58]. Standardized protocols for sampling, background controls, sample processing and analysis are non-existent [59,60,61], and those experimental details are often not included in scientific publications, further complicating a possible standardization of analytical methods.

## 4. Limitations in Study Comparability

Most studies evaluating biological effects in cell culture and animal models used pristine PS particles as mentioned above, but sizes differed over a huge range from nano- to micrometer scale, and some studies used particles with surface modifications (e.g., conjugated fluorophores [36,62,63] or chemical modifications such carboxyl groups [22,37]). Neither concentrations nor exposure times are in a comparable range. A plethora of mammalian model systems (e.g., cell lines Caco-2, A549, THP-1, peripheral blood monocytic cells, dermal fibroblasts; animal models like mouse and rat; recently reviewed in [64]) are used and an heterogenous set of readouts (e.g., cytotoxicity [28,32,63], secretion of inflammatory cytokines such as TNFα, IL-6 and IL-8 [22,39,65], generation of reactive oxygen species (ROS) [28,32,65], DNA damage [28,66]) are reported in the respective publications, complicating any attempt to attain an overall picture on the biological effects of NMPs, as also summarized in recently published reviews [56,64].

In general, there is a strong need for standardized reference materials to be used in comprehensive studies performed in mammalian experimental systems (including omics approaches), which systematically investigate and compare the effects of:

(a) NMPs made of the most abundantly produced polymers (PE, PP, PET, PVC, PU, PS);
(b) NMPs with different surface chemistry (i.e., pristine vs. weathered particles);
(c) NMPs with different sizes (nm–µm range) and shapes (spheres, fibers, agglomerates);
(d) NMPs of different chemical composition with common additives (i.e., leakage studies);
(e) NMPs with adsorbed biological material (i.e., with protein corona, microbial biofilm);
(f) NMPs applied at different concentrations and exposure times (acute vs. chronic effects).

In order to obtain a comprehensive overview on the human health risks posed by NMPs in our environment, novel reference materials must be established, reflecting the properties of “naturally” generated NMPs as precisely as possible. This includes the different physical and chemical properties, size distribution and surface modifications which ultimately determine the way in which NMPs interact and whether they are able to cross cellular membranes.

## 5. Future Assessments of the Human Health Risks of NMPs Could Be Improved

Future assessments of the human health risks of NMPs could be improved by:

(1) The production and distribution of standardized reference materials, ideally covering all major polymer types. Two promising approaches include the production of NMPs from plastic foils by laser ablation under aqueous conditions [32], which yields particles of defined size and physico-chemical surface characteristics similar to plastics degraded in the environment by UV irradiation and weathering. A complementary approach to laser ablation could be the coating of metallic nanoparticles with a polymeric surface. A major advantage of this approach would be that the metal core of such particles could be easily detected in biological samples, and the plastic coating could be treated by simulated weathering in order to reach a naturally degraded surface. In addition, the incorporation of fluorescent probes in the metal core of such particles would elegantly circumvent the widely neglected problem arising when reference particles are modified by conjugating fluorophores to the surface, thereby altering the particle’s surface chemistry.

Further developments in those two areas could lead to a panel of NMPs serving as a reference set used for standardized and comprehensive assessments of biological effects in various experimental models.

(2) A standardized sampling and sample processing strategy should be developed. NMPs are omnipresent, including laboratory facilities, and lead to a certain level of background contamination, which will differ between labs around the world. In addition, most lab equipment is made of plastic, itself emitting NMPs. Therefore, crucial issues for future studies include blank controls to determine the NMP background in the particular lab environment, and the replacement of plastic instrumentation and lab utilities with glass or steel (where available). A minimal set of guidelines should be defined based on a broad consensus in the scientific community, and recommended as a standard to be performed in assessment studies on NMPs, which identifies their chemical and surface properties, size and concentration. Interlaboratory tests are important to enable standardization and harmonization of sampling and analytical procedures.

In order to allow inter-study comparability, NMP exposures should follow a scheme including low (physiologically relevant levels), middle and high concentrations, applied for a few hours, several days, or the long term (if technically possible).

(3) The biological readout in mammalian cell culture models should include a minimal set of parameters such as cell viability/cytotoxicity (e.g., MTT, LDH release, colony forming efficiency), inflammatory cues (e.g., expression and release of TNFα and pro-inflammatory interleukins) and DNA damage (e.g., micronucleus test, Comet or TUNEL assay). An important aspect is the potential contamination of NMPs with endotoxins (e.g., lipopolysaccharides), which can elicit inflammatory reactions in cells. Both industrially produced NMPs as well as environmental samples might contain endotoxins, therefore pyrogen-free reference materials should be included as a control in such assays.

Furthermore, uptake of NMPs by cells must be documented, and advanced cell culture models should be established to test for NMPs passing through biological barriers, reflecting the postulated main uptake routes for NMPs: the airway and gastrointestinal tract. A commonly used, yet not ideal model, is the colorectal cancer cell line Caco-2, which is used to study the gastrointestinal barrier, but also more complex models combining different cell types should be implemented, as uptake of NMPs is specific to certain cells and depend on size, charge and other NMP characteristics [67,68,69].

In order to experimentally recapitulate the NMP uptake route via inhalation, co-cultures of different cell types, e.g., building up the nose mucosa and lung epithelium, should be envisaged [70,71]. An example of such a model is the pulmonary coculture system representing the lung–blood barrier, using human bronchial epithelial cells (16HBE14o-), monocytes (THP-1) and human lung microvascular endothelial cells (HLMVEC) [72]. Particles in the submicron range and, in particular, nanoplastics below 100 nm are able to penetrate cell membranes, possibly reach the blood stream and could be distributed throughout the organism, as reported in a rat model [68]. This raises the question of whether NMPs could also penetrate the blood–brain barrier, accumulate in the brain and elicit neurotoxic effects [73].

NMPs were shown to alter the gut microbiome in mice [74,75,76] and larval zebrafish [77], hence unexpected effects of NMPs via the microbiota-gut-brain axis [78,79] on neurological functions are conceivable. An interaction and fast colonization of plastic particles by microbes in aquatic environments is already known [80,81,82,83], and it seems plausible that such colonized particles could transfer microbes (including pathogenic species) to organisms if ingested. Hence, NMPs could be both carriers of microbes, as well as substrate for (gut) microbiomes, affecting metabolic processes or altering microbiome composition leading to dysbiosis. Clearly, NMP exposure is not limited to the gut and might impact not only the whole human microbiome (e.g., lung, skin) but also health-relevant environmental microbiomes. Studies should, therefore, consider NMPs pre-incubated with microbial cultures to mimic environmental bacteria/NMP interactions.

## 6. Concluding Remarks

In past years the topic “microplastics pollution and possible health risks for humans” raised much attention in the public, and led to political discussions to limit or ban the industrial use of plastic microparticles in various products, although scientific data proving or disproving acute or long-term toxicity of NMPs are scarce. Owing to the fact that it is extremely difficult to isolate and (chemically) identify micro- and, in particular, nanoparticles from environmental and biological samples, precise numbers about the human exposure towards NMPs via the supposedly main uptake routes of ingestion and inhalation have not been determined yet. Only one study has been successful so far in detecting microplastics in humans (stool samples) [84] and could roughly estimate the average exposure. Effects on human health remain elusive. The facts that global plastics production has been growing exponentially since the 1950s, and that nearly the entire human population is nowadays permanently exposed to plastic-derived NMPs, warrant a critical view of possible health risks posed by NMPs, likely not arising from acute toxicity but maybe from long-term accumulation in the human body, or indirect effects caused by alterations in the gut microbiome. In order to detect low-level effects in laboratory model systems, procedures and reference materials must be highly standardized, well defined and characterized, to allow well-grounded conclusions and decisions based on scientifically sound data.
